# Tackling Drug Resistance and Virulence in *Proteus mirabilis* Infections: Evaluating Rosemary Extract as a Complementary Therapeutic Agent

**DOI:** 10.1155/cjid/8300509

**Published:** 2026-08-29

**Authors:** Arezoo Mirzaei, Jeroen Wagemans, Sharareh Moghim

**Affiliations:** ^1^ Department of Bacteriology and Virology, School of Medicine, Isfahan University of Medical Sciences, Isfahan, Iran, mui.ac.ir; ^2^ Department of Biosystems, KU Leuven, Leuven, Belgium, kuleuven.be

**Keywords:** carbapenemase, *Proteus mirabilis*, *Rosmarinus officinalis L.*, biofilm, swarming

## Abstract

**Background and Aims:**

The rising antimicrobial resistance in *Proteus mirabilis*, a primary contributor to catheter‐associated urinary tract infections, underscores the urgent need to explore alternative therapeutic approaches. In this study, we evaluated the antibacterial and antivirulence potential of *Rosmarinus officinalis* L. extract (ROE) against *P. mirabilis* and concurrently assessed clinical isolates for the presence of key resistance and virulence genes.

**Methods:**

Forty multi–drug‐resistant (MDR) *P. mirabilis* isolates were analyzed using multiplex PCR to detect carbapenemase genes (*b* *l* *a*_NDM_, *b* *l* *a*_OXA_ family, *b* *l* *a*_SIM−1_, *b* *l* *a*_VIM−1_) and uniplex PCR to identify virulence determinants (*mrp*A, *pmf*A, *rsb*A, *rsm*A, *lux*S). ROE was extracted and analyzed using high‐performance liquid chromatography (HPLC). The antibacterial, antibiofilm, and antiswarming effects of the extract were assessed.

**Results:**

All isolates carried all five virulence genes. Carbapenemase gene profiling revealed *b* *l* *a*_VIM−1_in 20% of isolates, while *b* *l* *a*_OXA−23_, *b* *l* *a*_OXA−24_, and *b* *l* *a*_OXA−58_ were detected in 10%, 2.5%, and 2.5% of isolates, respectively. Neither *b* *l* *a*_SIM−1_ nor *b* *l* *a*_NDM_ was identified. HPLC analysis of ROE identified rosmarinic acid as the predominant compound. Although the agar well diffusion assay indicated no bactericidal activity, the broth microdilution method determined a minimum inhibitory concentration (MIC) of 8 mg/mL for both reference and MDR strains. At sub‐MIC levels, ROE significantly inhibited swarming motility (up to 86% at 250 μg/mL) and biofilm formation (up to 78% in the reference strain and 76% in MDR isolates at 15.6 μg/mL) in a dose‐dependent manner. Notably, biofilm eradication activity was observed only against preformed biofilms of the reference strain, achieving up to 53% reduction. MTT cytotoxicity assays confirmed that ROE in low concentrations (1000‐15.6 μg/mL) was nontoxic to Vero cells.

**Conclusion:**

These results highlight the concerning prevalence of carbapenemase genes among clinical *P. mirabilis* isolates and identify ROE as a promising, nontoxic candidate for targeting motility and biofilm formation. These findings suggest ROE may offer a viable strategy for managing drug‐resistant *P. mirabilis* infections.

## 1. Introduction

Catheter‐associated urinary tract infections (CAUTIs) are among the most common healthcare‐acquired infections and are strongly associated with the use of indwelling urinary catheters. These devices facilitate bacterial entry into the bladder during insertion, through the catheter lumen or by ascending along the catheter–urethral interface. Even short‐term catheterization may lead to infection, while prolonged catheterization is commonly associated with bacteriuria and recurrent complications such as catheter blockage. Among the causative pathogens, *Proteus mirabilis* is particularly important due to its ability to colonize the urinary tract and contribute to nosocomial infections [1, 2].

After the initial colonization of the catheter surface, *Proteus* species, along with other uropathogens, form distinctive crystalline biofilm structures during CAUTIs. While standard biofilms consist of soft, organic matrices that cause localized infections, crystalline biofilms are rigid, mineralized structures produced by urease‐positive bacteria and present a distinct clinical challenge due to their ability to rapidly obstruct medical devices [3].

These structures facilitate the persistence of *P. mirabilis* in the urinary tract by protecting the organism from antibiotics and the host immune response, while also promoting surface adhesion [4]. The formation of urinary stones is a hallmark of *Proteus*‐mediated urinary tract infections and plays a crucial role in the development of crystalline biofilms. These bacteria‐derived stones account for up to 30% of all urinary tract stones worldwide and approximately 75% of those classified as staghorn calculi [5]. Stones that form in the renal pelvis and extend into at least two calyces are classified as staghorn calculi, a type of upper urinary tract stone [6]. During CAUTIs, crystalline biofilms can be particularly problematic, as they can cause encrustation and lead to catheter blockages.

Recent studies have shown that *P. mirabilis* is highly associated with multidrug resistance (MDR), primarily due to the accumulation of both intrinsic and acquired antimicrobial resistance (AMR) genes. Genomic investigations have identified resistance genes associated with several major antibiotic classes, including *β*‐lactams, fluoroquinolones, aminoglycosides, sulfonamides, and carbapenems, suggesting that this species is emerging as an important reservoir of clinically relevant resistance determinants. These findings highlight the need for continued surveillance and emphasize the clinical significance of *P. mirabilis* in the era of global AMR [7].

A wide range of medicinal plants exists in Iran, along with biodiversity, as traditional medical practices incorporate various native herbs [8]. Rosemary stands as one of the most popular medicinal plants because it demonstrates active properties, including antioxidant, antimicrobial, and anti‐inflammatory activities [9]. Rosemary, as part of the Lamiaceae family, includes more than 7000 species worldwide [10]. Rosemary (*Rosmarinus officinalis*) is a perennial plant that is of special importance in Iran due to its wide use in the pharmaceutical and health industries [11]. The reduced effectiveness of conventional antimicrobial agents, combined with the growing prevalence of antibiotic resistance, remains a significant global healthcare challenge [12]. *R. officinalis* L. possesses a rich composition of bioactive compounds that might serve as an alternative medical treatment option [13]. Preventing and managing CAUTIs is a crucial aspect of infection control in healthcare settings [14], and understanding the antibacterial properties of *R. officinalis* extract (ROE) against *P. mirabilis* in these infections is important for developing effective prevention and treatment strategies.

Considerable evidence exists regarding the antimicrobial properties of rosemary (*R. officinalis* L.) extract against various pathogenic microorganisms. These effects are largely attributed to its rich phenolic content, including carnosic acid and rosmarinic acid. For instance, numerous studies have demonstrated its significant inhibitory effects against Gram‐positive bacteria like *Staphylococcus aureus*, including methicillin‐resistant strains (MRSA), where it has been shown to disrupt cell membrane integrity and inhibit biofilm formation [15–17]. Similarly, its activity has been reported against key Gram‐negative pathogens, such as *Escherichia coli* and *Pseudomonas aeruginosa* [18–20]. In these cases, the extract’s components have been found to interfere with crucial bacterial processes like quorum sensing and virulence factor production [21, 22]. Beyond clinical pathogens, its effectiveness against foodborne bacteria such as *Listeria monocytogenes* and *Salmonella* spp. is well established, supporting its potential use as a natural food preservative [23–26]. While these findings underscore the potential of rosemary as a source of antimicrobial agents, its specific activity against *P. mirabilis* and its impact on the unique challenge of crystalline biofilm formation remain significantly underexplored.

*R. officinalis* was selected for this study for several reasons. First, the plant exhibits broad‐spectrum activity against both Gram‐positive and Gram‐negative bacteria; hence, the current research could be considered promising in investigating the effect of the selected plant extract against *P. mirabilis*. Second, the well‐characterized nature of its primary bioactive compounds (carnosic acid, rosmarinic acid) provides a strong foundation for future mechanistic studies. Finally, rosemary’s favorable safety profile, wide availability, and extensive use in traditional medicine for treating infections further support its selection as a practical and relevant natural product for developing innovative treatment approaches. Therefore, we hypothesized that the potent antibiofilm and antimicrobial properties reported in the literature could be effectively leveraged to combat the formation of crystalline biofilms, a clinically persistent problem requiring new solutions.

This investigation evaluates the antibacterial properties of *R. officinalis* L., a plant with established ethnopharmacological relevance, in the context of treating infections caused by *P. mirabilis*. In parallel, the study examines the prevalence of key genetic determinants, including the carbapenemase‐encoding gene *b* *l* *a*_NDM_, along with virulence‐associated genes *mrp*A, *pmf*A, *rsb*A, *rsm*A, and *lux*S. Molecular screening was conducted using multiplex polymerase chain reaction (PCR) assays targeting genes of the *b* *l* *a*_OXA_ family, as well as multiplex PCR specific for the metallo‐β‐lactamase genes *b* *l* *a*_SIM−1_ and *b* *l* *a*_VIM−1_.

## 2. Material and Methods

### 2.1. Isolation of Bacteria From the Urinary Catheter

This cross‐sectional study was conducted from June 2019 to July 2020 across multiple hospitals in Isfahan, Iran, where 385 nonduplicate catheters (indwelling for 5–15 days) were collected from intensive care unit patients. For bacterial isolation, catheters were sectioned into 1‐cm segments and first immersed in phosphate‐buffered saline (PBS) and washed three times to dislodge loosely adherent planktonic cells and reduce potential environmental contaminants. The segments were then transferred to 10 mL of PBS and subjected to vigorous vortexing for 1 min, followed by probe sonication at 10 W (RMS) for 60–90 s and a second round of high‐speed vortexing for 1 min [27, 28]. The resulting suspension was plated onto blood agar, eosin methylene blue (EMB) agar, and MacConkey agar, with isolates identified using standard biochemical tests. Species confirmation as *P. mirabilis* was achieved through PCR amplification of the *ure*G gene [29].

### 2.2. Antibiotic Susceptibility Testing

Antimicrobial susceptibility of the isolates was assessed using the disk diffusion method on Mueller–Hinton agar following an overnight incubation at 37^∘^C. The antibiotics tested included ampicillin–sulbactam (10/10 μg), amoxicillin–clavulanic acid (20/10 μg), ampicillin (10 μg), nitrofurantoin (300 μg), meropenem (10 μg), cefotaxime (30 μg), ceftazidime (30 μg), ceftazidime–clavulanic acid (30/10 μg), cefixime (5 μg), aztreonam (30 μg), amikacin (30 μg), norfloxacin (10 μg), ofloxacin (5 μg), ciprofloxacin (5 μg), tetracycline (30 μg), and trimethoprim–sulfamethoxazole (1.25/23.75 μg). Interpretation of inhibition zone diameters was performed in accordance with the Clinical and Laboratory Standards Institute (CLSI 2025) guidelines. MDR was defined based on the criteria proposed by Magiorakos et al. whereby isolates exhibiting resistance to at least one agent in three or more antimicrobial classes were categorized as MDR [30].

### 2.3. Biofilm Formation Assay

Overnight cultures of *P. mirabilis* isolates were diluted at a ratio of 1:100 in tryptic soy broth (TSB) to achieve a turbidity equivalent to 0.5 McFarland standard. Subsequently, 5 μL of the prepared suspension was inoculated into each well of sterile 96‐well polystyrene microtiter plates (Greiner, Germany). The plates were incubated at 37°C for 24 h to allow biofilm formation. Following incubation, the culture medium was carefully discarded, and the wells were gently rinsed with double‐distilled water (DDW) to remove nonadherent cells. The attached biofilms were then fixed using 200 μL of 96% ethanol for 15 min. After removing the ethanol, the plates were left to air dry. The fixed biofilms were stained with 0.1% (w/v) crystal violet solution for 20 min at 37°C. Excess stain was removed by washing the wells three times with DDW. To quantify biofilm formation, 200 μL of an ethanol–acetic acid solution (90:10) was added to each well to solubilize the bound dye. The optical density (OD) was then measured at 600 nm using an ELISA microplate reader. All experiments were performed in triplicate, and results were expressed as mean absorbance values ± standard deviation. *Escherichia coli* K12 and *Pseudomonas aeruginosa* ATCC 27853 were included as negative (weak biofilm producer) and positive (strong biofilm producer) control strains, respectively [31]. Based on this metric, isolates were categorized as follows: nonbiofilm formers (OD ≤ ODc), weak biofilm formers (ODc < OD ≤ 2 × ODc), moderate biofilm formers (2 × ODc < OD ≤ 4 × ODc), or strong biofilm formers (OD > 4 × ODc) [32].

### 2.4. PCR Amplification of *mrp*A, *pmf*A, *rsb*A, *rsm*A, *lux*S, and *b* *l* *a*_NDM_

The virulence‐associated genes involved in adhesion, biofilm formation, and regulatory functions, as well as the carbapenemase‐encoding gene *b* *l* *a*_NDM_ associated with resistance to *β*‐lactam antibiotics, were detected in *P. mirabilis* isolates using PCR.

DNA was extracted using the phenol–chloroform method as outlined by Sambrook et al. [33]. The quality and purity of the extracted DNA were assessed using a NanoDrop spectrophotometer (OD260/OD280 nm ratio ≥ 1.8). PCR conditions for all genes, except *rsm*A, were as follows: initial denaturation at 95°C for 3 min; 30 cycles of denaturation at 95°C for 30 s, annealing at 54.3°C for 30 s, and extension at 72°C for 1 min; and a final extension at 72°C for 5 min. The annealing temperature of the *rsm*A gene was 58°C [34]. The primers used in this study are listed in Table 1.

**TABLE 1:** The list of primers used in this study.

Gene	Primer sequence (5 ′–3 ′)	Product length	Reference
*b* *l* *a*_*O* *X* *A*23_	F: GATCGGATTGGAGAACCAGA R: ATTTCTGACCGCATTTCCAT	501	[35]

*b* *l* *a*_*O* *X* *A*24_	F: GGTTAGTTGGCCCCCTTAAA R:AGTTGAGCGAAAAGGGGAT	246	[35]

*b* *l* *a*_*O* *X* *A*58_	F: AAGTATTGGGGCTTGTGCTG R: CCCCTCTGCGCTCTACATAC	599	[35]

*b* *l* *a*_*V* *I* *M*_	F: GATGGTGTTTGGTCGCATA R: CGAATGCGCAGCACCAG	390	[36]

*b* *l* *a*_*S* *I* *M*_	F: TACAAGGGATTCGGCATCG R: TAATGGCCTGTTCCCATGTG	570	[36]

*b* *l* *a*_*N* *D* *M*_	F: CACCTCATGTTTGAATTCGCC R: CTCTGTCACATCGAAATCGC	982	[37]

*mrpA*	F: TGCTGCATTAAAAGATGGTGGC R: TTTGTTTACCACCCGCATCG	200	[34]

*pmfA*	F: GGCTGCGGCTTTAGTATTTG R: GGCTTGAAGATGCTGCTAATC	146	[34]

*luxS*	F: AAAGCCATGCCTGAGAAAGG R: CGACATCCCATTGGCGAAATA	116	[34]

*rsmA*	F: AGCCTTTAATCAGCGCCGTA R: GCGTGTTGCTGTTGTGATGA	165	[34]

*rsbA*	F: CGCTATCACGCTAACCAACTA R: GCGTCCTTCAAGCCAATAAAC	120	[34]

*ureG*	F: AGAATATAATCAACCACTGCGTA R: CATTTTGGCTGTATCCGCTTC	514	[38]

### 2.5. Multiplex PCR of *b* *l* *a*_OXA_ Family and Multiplex PCR of *b* *l* *a*_SIM−1_ and *b* *l* *a*_VIM1_

Three primer sets for multiplex PCR of the *b* *l* *a*_OXA_ family were used to amplify the internal region of the *b* *l* *a*_OXA58_, *b* *l* *a*_OXA23_, and *b* *l* *a*_OXA24_ genes; two primer sets for multiplex PCR of *b* *l* *a*_SIM−1_ and *b* *l* *a*_VIM1_ are shown in Table 1. The PCR conditions for all these genes were as follows: 3 min at 94°C, 30 s at 94°C, 30 s at 60°C, and 30 s at 72°C for 30 cycles, followed by a final extension of 5 min at 72°C. The amplicons were visualized by electrophoresis [39].

### 2.6. Preparation of ROE

The aerial parts of *Rosmarinus officinalis* L. were sourced from the botanical garden of the School of Pharmacy, Department of Pharmacognosy, at Isfahan University of Medical Sciences, Iran. Voucher specimen authentication was performed by a qualified botanist. A sample of freshly powdered plant material (100 g) underwent triple sequential extraction, each cycle consisting of 15 min (3 × 5 min) in 100 mL of 50% aqueous ethanol. The process was conducted under ice‐cooling using a rotor–stator homogenizer (Ultraturrax) at maximum operational speed. Following extraction, the combined suspensions were centrifuged at 5000 × g for 15 min. The resulting clear supernatant was subsequently concentrated using rotary evaporation under reduced pressure, yielding 2.0 g of a dried extract, corresponding to a 5:1 ratio of crude herb to extract [40].

### 2.7. High‐Performance Liquid Chromatographic (HPLC)

The active phytochemicals in the aqueous aerial extract were quantified via HPLC. Following acid hydrolysis of the dried extract (100 mg in 2.5 M HCl/tetrahydrofuran), flavonoid analytes were extracted via liquid–liquid partitioning using a 2 N HCl/diethyl ether system enhanced by salting‐out. Separation was achieved on a NUCLEOSIL RP‐18 column using an isocratic mobile phase of 10 mM phosphoric acid and acetonitrile (75:25 v/v) at 1.2 mL/min, with UV detection (200–500 nm). Quantification was performed using an external calibration curve (0.005–0.1 mg/mL), with analytes identified by retention time matching to standards. The concentration–peak area relationship was modeled by linear least‐squares regression [41].

### 2.8. Antimicrobial Activity of ROE by Agar Well Diffusion Assay

For initial antibacterial screening, the agar well diffusion assay was selected. The procedure began by preparing a stock solution of ROE in distilled water, followed by twofold serial dilutions to create a concentration range from 8000 to 15.6 μg/mL. Bacterial suspensions were standardized to approximately 1.5 × 10^8^ CFU/mL from overnight cultures and uniformly swabbed onto Mueller–Hinton agar (MHA) plates. Wells were then created in the inoculated agar using a sterile Pasteur pipette with a diameter of 6 mm, and 50 μL of each ROE concentration was carefully transferred into the respective wells. On each plate, wells containing cotrimoxazole (1.25/23.75 μg) and imipenem (10 μg) were included as standardized positive controls, alongside a negative control well filled with sterile distilled water. After an 18‐h–24‐h incubation, the clear zones of inhibition were examined and measured [42].

### 2.9. Minimum Inhibitory Concentration (MIC) of ROE

The MIC of ROE was assessed via broth microdilution in a 96‐well microtiter plate. Serial dilutions of ROE (16000  μg/mL–62.5  μg/mL) were prepared in Mueller–Hinton broth (MHB). Subsequently, each well was inoculated with a bacterial suspension to a final concentration of 10^5^ CFU/mL, using the reference strain *P. mirabilis* ATCC 7002 and clinically selected MDR strains. Following an 18‐ to 24‐h incubation period, the MIC was defined as the lowest concentration of ROE at which no visible bacterial growth was observed [43, 44].

### 2.10. Antiswarming Assay of ROE

The antiswarming activity of ROE against *P. mirabilis* was evaluated using an agar incorporation assay. For each test concentration, 50 μL of each serial dilution of ROE (1000–62.5 μg/mL) was aseptically mixed with 15 mL of MHA, which had been maintained in a molten state at 45°C. The ROE‐supplemented agar was immediately poured into a Petri dish and left to solidify at room temperature. Following solidification, a 5‐μL inoculum of *P. mirabilis* ATCC7002 (10^8^ CFU/mL) culture was deposited as a central spot on the agar surface. The plates were incubated aerobically at 37°C for 24 h–48 h. Postincubation, swarming motility was quantified by measuring the diameter of the bacterial migration zone, with inhibition expressed relative to growth on control plates containing no ROE [44, 45]. Antiswarming activity was calculated using the following formula:

(1) swarming inhibition%=swarming diameter in unsupplemented agar−swarming diameter in agar supplemented with ROEswarming diameter in unsupplemented agar×100.

### 2.11. Biofilm Inhibition and Biofilm Eradication Assay of ROE

ROE was evaluated for both biofilm inhibition and biofilm eradication activity against the reference strain *P. mirabilis* ATCC 7002 and selected MDR strong biofilm‐producing clinical isolates. For the biofilm inhibition assay, a standardized suspension of each bacterial strain (final concentration: 1.5 × 10^8^ CFU/mL) was coincubated with serial twofold dilutions of ROE in TSB within a 96‐well microtiter plate. The plate was incubated statically for 24 h at 37°C. Controls consisted of a positive control for each isolate, containing bacteria without ROE, and a negative control containing sterile TSB only [31]. All tests were performed in triplicate.

For the biofilm eradication assay, mature biofilms were first established by incubating bacterial suspensions in TSB for 24 h at 37°C. The planktonic cells were then removed, and the adherent biofilm was carefully rinsed. Fresh TSB containing serial dilutions of ROE was added to the preformed biofilms, followed by a further 24‐h incubation at 37°C.

For both assays, biofilm biomass was quantified by crystal violet staining. Following incubation, the contents of the wells were discarded, and the wells were gently rinsed with PBS to remove nonadherent cells. Biofilms were fixed with 96% ethanol for 15 min. After air‐drying, the attached biomass was stained with 0.1% (w/v) crystal violet for 10 min. Excess stain was removed by thoroughly washing the plate five times with distilled water. The bound dye was then solubilized with 200 μL of an ethanol/acetone (80:20 v/v) solution, and the OD of the resulting solution was measured at 600 nm using a microplate (ELISA) reader.

Biofilm inhibition and eradication efficacy were quantified and expressed as a percentage. The value was derived from the following standard formula [46]:

(2) % Inhibition or eradication=ODpositive ontrol−ODtest/ ODpositive ontrol×100.

### 2.12. Cytotoxicity Assay by MTT

The Vero cell line (ATCC CCL‐81) was cultured in 96‐well microtiter plates at an initial density of 5 × 10^3^ cells/well in DMEM supplemented with 5% FBS and incubated for 16 h under standard conditions (37°C, 5% CO2). A stock solution of ROE (16 mg/mL) was prepared and subjected to twofold serial dilutions in DMEM to achieve a test concentration range of 8000–15.6 μg/mL. Cells were exposed to 100 μL of each dilution for 24 h, with untreated cells serving as the growth control. Subsequent cell viability was determined via MTT assay according to an established protocol [47].

### 2.13. Statistical Analysis

Statistical analysis was performed using GraphPad Prism (Version 9). One‐way ANOVA, one‐sample *t*‐test, and Wilcoxon test were used to determine any statistical association. Statistical significance was regarded as *p* < 0.05.

## 3. Results

### 3.1. Bacterial Characterization and Antibiotic Susceptibility Testing

A total of 40 *P. mirabilis* isolates were recovered from urinary catheters. Most *P. mirabilis* isolates (72.5%) were recovered from female patients, with a median patient age of 42.5 years. Isolates from longer catheterization periods generally showed stronger biofilm formation, while those from shorter catheterization periods tended to be weaker biofilm producers.

Among these isolates, the highest resistance rates were observed against tetracycline (95%), followed by nitrofurantoin (92.5%), trimethoprim/sulfamethoxazole (75%), ciprofloxacin (45%), cefotaxime (42.5%), ofloxacin (40%), ampicillin (35%), meropenem (30%), norfloxacin (25%), cefixime and amoxicillin–clavulanate (22.5%), aztreonam and amikacin (15%), ceftazidime (7.5%), and ampicillin–sulbactam (2.5%).

Based on the criteria proposed by Magiorakos et al., isolates resistant to at least one agent in three or more antimicrobial classes were classified as MDR; accordingly, 82.5% of the 40 isolates were identified as MDR.

### 3.2. Biofilm Formation of Isolates

All *P. mirabilis* isolates recovered from catheters were capable of biofilm formation. Among the 40 isolates, 12 (30%) exhibited strong biofilm production, 20 (50%) were classified as moderate biofilm producers, and 8 (20%) showed weak biofilm formation.

### 3.3. PCR Amplification of *b* *l* *a*_NDM_, *mrpA*, *pmfA*, *rsbA*, *rsmA*, and *luxS*, *and* Multiplex PCR of *b* *l* *a*_OXA_ Family, *b* *l* *a*_*S* *I* *M*−1_, and *b* *l* *a*_VIM1_ *.*

Among the isolates recovered from catheters, the *b* *l* *a*_NDM_ gene was not detected. The frequencies of *b* *l* *a*_OXA23_, *b* *l* *a*_OXA24_, and *b* *l* *a*_OXA58_ were 10% (4/40), 2.5% (1/40), and 2.5% (1/40), respectively, with some isolates simultaneously positive for three *b* *l* *a*_OXA_ genes.

Multiplex PCR for *b* *l* *a*_SIM−1_ and *b* *l* *a*_VIM−1_ revealed that 20% (8/40) of the isolates were positive for *b* *l* *a*_VIM−1_, while *b* *l* *a*_SIM−1_ was not detected in any isolate; co‐carriage of both genes was also absent. There was no significant relationship between the presence of capsular genes and biofilm formation. All isolates (100%) were positive for *mrp*A, *pmf*A, *rsb*A, *rsm*A, and *lux*S.

### 3.4. HPLC of ROE

**FIGURE 1:**
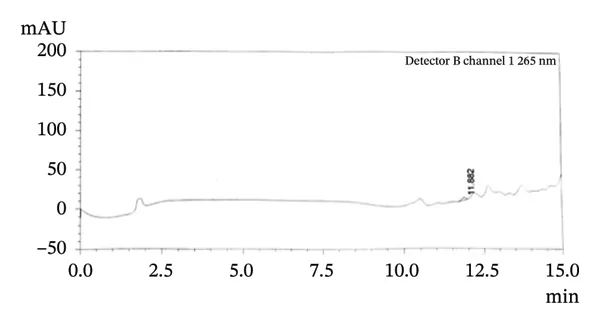
HPLC results of crude extract of *Rosmarinus officinalis* L.

Chromatographic analysis revealed a distinct and well‐resolved peak at a retention time of 11.882 min. By comparing the retention time and spectral characteristics of this peak with those of the available rosmarinic acid standard, it was confidently identified as rosmarinic acid (Figure 1). Although the presence of additional phytochemical constituents in the extract is highly likely, their identification could not be confirmed due to the lack of corresponding analytical standards. Nevertheless, given the reported prevalence of rosmarinic acid in rosemary and its known biological activities, this compound was considered a key component for the standardization and interpretation of the extract’s bioactivity.

The dried extract was therefore standardized using rosmarinic acid as the bioactive marker (Figure 1).

### 3.5. Antimicrobial Activity of ROE by Agar Well Diffusion Assay and MIC

No bactericidal activity was observed for ROE against either the reference strain *P. mirabilis* ATCC 7002 or the MDR clinical isolates in the agar well diffusion assay, as indicated by the absence of any zone of inhibition (data not shown). Conversely, the broth microdilution method revealed an inhibitory effect, with the MIC determined to be 8 mg/mL for both the reference strain and the MDR isolates.

### 3.6. Antiswarming Assay of ROE

**FIGURE 2:**
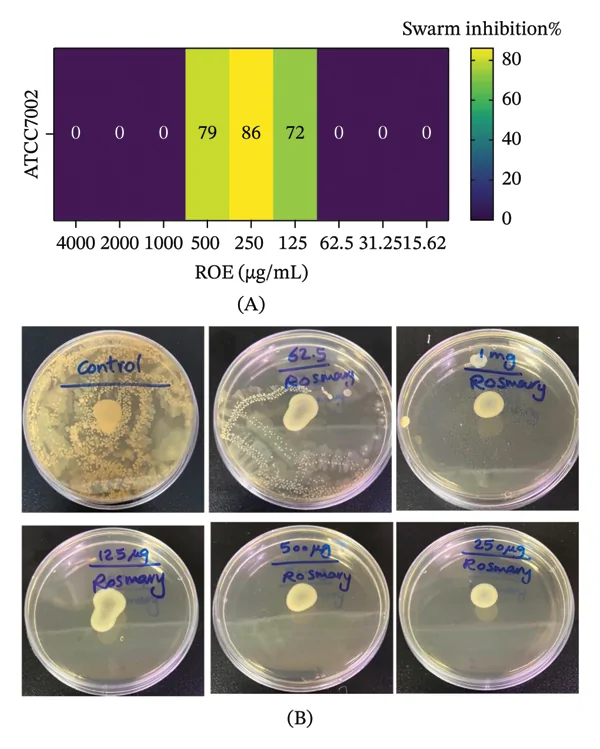
The antiswarming activity of ROE was evaluated against *P. mirabilis* ATCC 7002. Subinhibitory concentrations (4000–15.6 μg/mL) of ROE exhibited a dose‐dependent suppression of swarming motility, with inhibition ranging from 0% to 86%. The highest inhibition (86%) was observed at 250 μg/mL. (A) Quantitative analysis of swarm inhibition at varying concentrations of ROE. No significant inhibitory effect was detected at the extreme concentrations (4000 μg/mL and 15.6 μg/mL). Statistical analysis using the one‐sample *t*‐test and Wilcoxon signed‐rank test confirmed significant inhibition at 500, 250, and 125 μg/mL (*p* = 0.0002, 0.0002, and 0.0006, respectively). (B) Representative images showing the pattern of motility inhibition on agar plates.

Sub‐MICs of ROE, ranging from 4000 to 15.6 μg/mL, were evaluated for their ability to suppress the swarming motility of *P. mirabilis* ATCC 7002. Inhibition occurred in a dose‐dependent manner, with efficacy varying between 0% and 86% across the concentration gradient. The optimal antiswarming activity was observed at 250 μg/mL, which yielded 86% inhibition. No significant inhibitory effect was detected at the extreme concentrations (4000 μg/mL and 15.6 μg/mL) of the tested spectrum (Figure 2). Statistical validation using the one‐sample *t*‐test and Wilcoxon signed‐rank test confirmed that inhibition was statistically significant at concentrations of 500, 250, and 125 μg/mL, yielding *p*‐values of 0.0002, 0.0002, and 0.0006, respectively.

### 3.7. Biofilm Inhibition and Biofilm Eradication Assay of ROE

**FIGURE 3:**
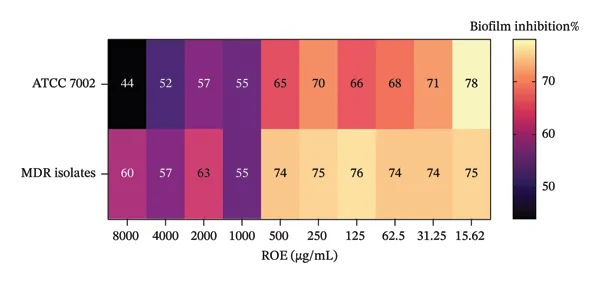
Percentage of biofilm inhibition by ROE at different concentrations against MDR clinical isolates and ATCC 7002 strain of *P. mirabilis.* ROE demonstrated significant inhibitory activity in a dose‐dependent manner across all tested strains. The optimal inhibitory concentration was determined to be 15.6 μg/mL, at which biofilm formation was reduced by 44%–78% in the reference strain and 55%–76% in MDR isolates. Statistical analysis using one‐way ANOVA revealed that biofilm inhibition at 15.6 μg/mL was significantly greater compared to other tested concentrations (*p* < 0.04; 95% CI). Data are presented as mean ± standard deviation.

ROE exhibited significant, dose‐dependent inhibition of biofilm formation in both MDR clinical isolates and the reference strain *P. mirabilis* ATCC7002. The optimal inhibitory concentration was 15.6 μg/mL for all tested strains. Biofilm inhibition ranged from 44% to 78% for the reference strain (ATCC 7002) and from 55% to 76% for the MDR isolates (Figure 3). Statistical analysis using one‐way ANOVA showed that the biofilm inhibition observed at 15.6 μg/mL was significantly different from that at the other tested concentrations (*p* < 0.04, 95% confidence interval).

**FIGURE 4:**
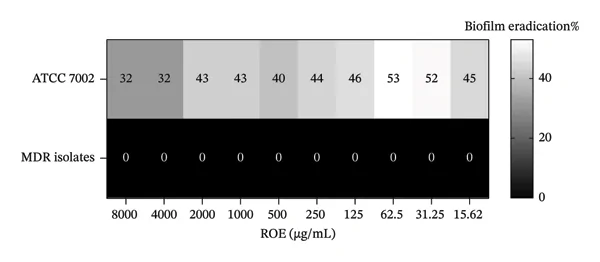
Percentage of biofilm eradication by ROE at different concentrations against MDR clinical isolates and ATCC 7002 strain of *P. mirabilis*. ROE exhibited biofilm eradication activity only against the reference strain, showing a dose‐dependent reduction ranging from 32% to 53%, with the highest effect observed at 62.5 μg/mL. No significant eradication of preformed biofilms was detected in MDR isolates under the tested conditions. One‐way ANOVA revealed no statistically significant differences (*p* > 0.99999, 95% CI) between concentrations for either MDR isolates or the reference strain.

Regarding biofilm eradication, ROE demonstrated activity only against the reference strain. Treatment resulted in a dose‐dependent eradication of 32% to 53%, with an optimal concentration of 62.5 μg/mL. No significant eradication of preformed biofilms was observed for the MDR isolates under the tested conditions (Figure 4). One‐way ANOVA indicated that there was no statistically significant relationship (*p* > 0.99999, 95% CI) between the tested concentrations and biofilm eradication in either MDR isolates or the reference strains.

### 3.8. Cytotoxicity Assay by MTT

**FIGURE 5:**
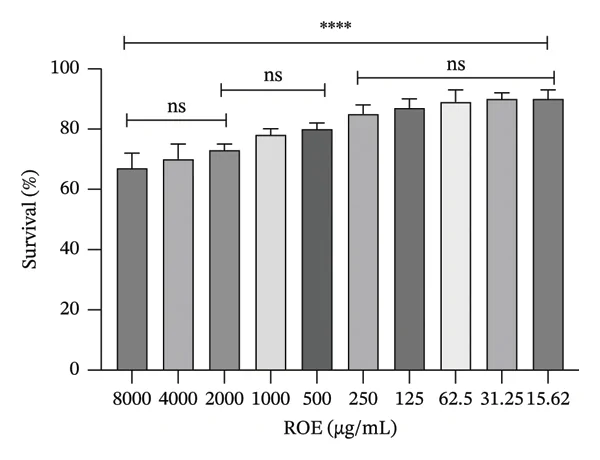
Cell cytotoxicity using an MTT assay following treatment with different concentrations of ROE, with absorbance measured at 570 nm. One‐way ANOVA indicated no significant reduction in cell viability at lower concentrations (500–15.6 μg/mL; *p* > 0.05), suggesting noncytotoxicity within this range. In contrast, a significant decrease in cell viability was observed at higher concentrations compared to the lower concentration range (*p* < 0.05).

Cytotoxicity of ROE was assessed in Vero cells using the MTT assay following 24‐h exposure to concentrations ranging from 8000 to 15.6 μg/mL. Statistical analysis revealed no significant reduction in cell viability at lower concentrations (500–15.6 μg/mL; *p* > 0.05), indicating that ROE is noncytotoxic within this range under the tested conditions (Figure 5). However, a significant decrease in cell viability was observed at higher concentrations compared to the lower concentration range.

## 4. Discussion

CAUTIs represent a major clinical burden among healthcare‐associated infections, with *P. mirabilis* emerging as a principal causative pathogen owing to its urease activity, swarming motility, and robust biofilm‐forming capacity. The ability of urease‐positive strains to promote crystalline biofilm formation and urinary stone development makes the eradication of infection particularly challenging, a problem further worsened by the escalating prevalence of AMR in catheter‐related settings [48, 49].

The dissemination of resistance in *P. mirabilis* is driven not only by mutational adaptation and selective pressure, but also by horizontal gene transfer, which facilitates the acquisition of resistance determinants from phylogenetically distinct organisms. In the present study, one isolate carried three OXA‐type carbapenemase genes, namely, OXA‐23, OXA‐24, and OXA‐58. This is noteworthy because these genes are classically associated with *A. baumannii* and their detection in *P. mirabilis* reflects the spread of major carbapenem resistance determinants beyond their traditional host species. These findings support the idea that hospital‐associated bacteria may act as shared genetic platforms for the circulation of resistance genes, thereby contributing to the broader AMR crisis in clinical environments. A previous study in 2022 also reported the detection of *b* *l* *a*_OXA−23_ in a *P. mirabilis* urinary isolate, further supporting the possibility of horizontal transfer between distinct Gram‐negative pathogens [50–52]. The detection of *b* *l* *a*_VIM−1_ in our isolates is equally concerning. Metallo‐β‐lactamases such as VIM enzymes are clinically important because they result in resistance to carbapenems, which are often used as last‐line therapy for severe infections. Although *b* *l* *a*_SIM−1_ and *b* *l* *a*_VIM−1_ are relatively uncommon in *P. mirabilis* compared to other organisms, their appearance in *P. mirabilis* has been increasingly documented in healthcare‐associated infections [53]. The absence of *b* *l* *a*_SIM−1_ and *b* *l* *a*_NDM_ in our study is consistent with their characteristically low prevalence in this species [54–59]. These findings are consistent with previous reports showing the increasing diversity of *β*‐lactamase genes in *P. mirabilis*. For instance, Beltrão et al. reported multiclonal dissemination and marked genetic variability among isolates, with frequent detection of *b* *l* *a*_*O* *X* *A*−10−*l* *i* *k* *e*_, *b* *l* *a*_KPC_, and *b* *l* *a*_NDM_, including the co‐carriage of resistance genes [60]. Similarly, Joseph et al. observed a high rate of multidrug resistance (83%) and identified *b* *l* *a*_NDM−1_ and *b* *l* *a*_KPC−2_, both of which were associated with resistance to multiple antibiotic classes. Shaaban et al. also demonstrated a strong association between phenotypic resistance and the presence of genes such as *b* *l* *a*_SHV_, *b* *l* *a*_AmpC_, and *b* *l* *a*_VIM−1_ [61]. Together, these studies support our observation that genotypic resistance determinants are generally reflected in phenotypic resistance patterns, although some variation may arise from differences in gene expression or regulatory control. The detection of *b* *l* *a*_OXA−23_, *b* *l* *a*_OXA−24_, *b* *l* *a*_OXA−58_, *b* *l* *a*_VIM−1_, and *b* *l* *a*_SIM−1_ is consistent with the observed phenotypic resistance to carbapenems, supporting their role in the reduced susceptibility of the isolates to *β*‐lactam antibiotics. However, no significant direct relationship was observed between the presence of individual genes and the phenotypic resistance profile, suggesting that the resistant phenotype likely results from the combined contribution and expression of multiple resistance determinants.

Overall, our findings highlight the importance of linking the molecular detection of resistance genes with phenotypic susceptibility profiles to better assess their clinical significance. Continuous local surveillance of carbapenemase genes in *P. mirabilis* is particularly important in catheter‐associated infections, where persistence and resistance often coexist. In response to these therapeutic limitations, growing scientific interest has focused on plant‐derived compounds as adjunctive approaches that target bacterial virulence rather than growth inhibition alone [62].

*R*. *officinalis* L. is a well‐characterized medicinal plant whose antimicrobial, antioxidant, and anti‐inflammatory properties are attributed to a complex mixture of phenolic acids, flavonoids, and diterpenoids, with rosmarinic acid, carnosic acid, and carnosol among its most biologically active constituents [9].

In the present study, HPLC analysis confirmed rosmarinic acid as the predominant detected compound; however, the overall biological activity of rosemary extract (ROE) is most likely due to synergistic interactions among multiple phytochemical constituents rather than the action of a single compound. The phytochemical profile and associated biological activity of rosemary extracts are inherently variable and are influenced by plant origin, harvest conditions, and extraction methodology [63].

A noteworthy finding of the present study is the absence of inhibitory activity in the agar well diffusion assay, despite demonstrable antivirulence effects in the biofilm and swarming assays. This discrepancy is common for crude extracts, particularly when active constituents are large, poorly diffusive, or present as complex mixtures. Agar diffusion‐based methods are inherently dependent on compound mobility through the matrix and may therefore fail to detect subinhibitory biological effects that are captured by virulence‐specific assays [64]. Likewise, the MIC of ROE was determined to be 8 mg/mL, indicating that high concentrations of the crude extract are required for direct antibacterial activity. At sub‐MIC levels, however, ROE significantly suppressed swarming motility and inhibited biofilm formation, consistent with other studies that confirm the predominantly antivirulence activity of the crude extract rather than a bactericidal mode of action [34].

Regarding biofilm inhibition, ROE demonstrated significant dose‐dependent activity against both the reference strain and MDR clinical isolates, with optimal inhibition observed at 15.6 μg/mL. The nonlinear dose–response pattern observed, wherein maximum inhibitory activity was recorded at the lowest tested concentration, warrants mechanistic consideration. This phenomenon, while atypical relative to classical pharmacological models, is not unprecedented in studies involving crude plant extracts and is likely attributable to the heterogeneous phytochemical composition of ROE [65]. At lower sub‐MIC concentrations, specific bioactive constituents, potentially including polyphenols, flavonoids, or tannins, may selectively interfere with quorum sensing signaling pathways and biofilm regulatory genes without exerting broad bactericidal pressure [66]. At higher concentrations, however, antagonistic interactions among co‐occurring phytochemical constituents may decrease the inhibitory effect [65]. Furthermore, increasing concentrations may trigger compensatory stress–response mechanisms in *P. mirabilis*, including the upregulation of efflux pumps or enhanced extracellular matrix production, which could surprisingly diminish the observed inhibitory efficacy [67]. Our study demonstrated that ROE effectively inhibited biofilm formation, but it did not eradicate preformed biofilms in MDR isolates, suggesting a predominantly preventive rather than curative antibiofilm capacity. The biological basis of this discrimination is consistent with the biology of developing biofilms. The dynamic processes of adhesion occur during the early stage of biofilm development and may be disrupted by quorum sensing; however, mature biofilm structures are complex, form an extracellular matrix, and have diminished permeability to antimicrobial agents. ROE therefore appears most efficacious as an agent targeting biofilm initiation rather than established biofilm eradication [68]. The antiswarming activity of ROE, which was also dose‐dependent, further supports an antivirulence mechanism of action. Swarming motility in *P. mirabilis* is essential for catheter colonization and ascending urinary tract infection, and its suppression, even partial, carries potential clinical significance. The fimbrial adhesins encoded by *mrp*A and *pmf*A are associated with surface colonization and urinary tract persistence; since swarming and adhesion are functionally interconnected in biofilm development, inhibition of motility may indirectly attenuate the bacterium’s capacity to initiate surface colonization. Although urease activity was not directly assessed in the present study, its role as a key virulence determinant in *P. mirabilis* facilitating urinary alkalinization, struvite stone formation, and catheter encrustation warrants future investigation into whether ROE modulates urease‐associated regulatory networks [4, 69, 70].

### 4.1. Limitation and Future Direction

Although our results confirm ROE as a promising phytochemical antivirulence agent against MDR *P. mirabilis*, in vivo studies and catheter‐based models are required to validate its efficacy under physiological conditions. Further molecular investigations, including analysis of gene expression related to adhesion and quorum sensing, given the high prevalence of these genes, will also help to determine how ROE alters virulence regulation and whether it can be developed as an adjunctive strategy for preventing CAUTIs. Additionally, our HPLC analysis detected only the major compound using a commercial standard for validation, potentially missing minor constituents due to the method’s sensitivity limitations.

## 5. Conclusion

In conclusion, ROE demonstrated its effects through multiple mechanisms, interfering with bacterial adhesion, decreasing swarming motility, and suppressing early biofilm development. This multifactorial mode of action reflects the complex phytochemical composition of the extract, in which rosmarinic acid likely plays an essential role, enhanced by synergistic interactions with co‐occurring phenolic and diterpenoid constituents. Although these findings position ROE as a promising antivirulence candidate for catheter‐associated *P. mirabilis* infections, confirming its therapeutic value will require targeted mechanistic work and in vivo studies.

## Funding

This research benefited from a grant from the Isfahan University of Medical Sciences. This work was supported by Isfahan University of Medical Sciences, Isfahan, I.R. Iran, through Grant No. 1401268.

## Conflicts of Interest

The authors declare no conflicts of interest.

## Data Availability

Data sharing is not applicable to this article as no datasets were generated or analyzed during the current study.
